# Interleukin-6 Induces Vascular Endothelial Growth Factor-C Expression via Src-FAK-STAT3 Signaling in Lymphatic Endothelial Cells

**DOI:** 10.1371/journal.pone.0158839

**Published:** 2016-07-06

**Authors:** Yu-Han Huang, Hung-Yu Yang, Shiu-Wen Huang, George Ou, Ya-Fen Hsu, Ming-Jen Hsu

**Affiliations:** 1 Graduate Institute of Medical Sciences, College of Medicine, Taipei Medical University, Taipei, Taiwan; 2 Division of Cardiovascular Medicine, Department of Internal Medicine, Taipei Medical University-Wan Fang Hospital, Taipei, Taiwan; 3 Department of Internal Medicine, School of Medicine, College of Medicine, Taipei Medical University, Taipei, Taiwan; 4 Graduate Institute of Biomedical Informatics, College of Medical Science and Technology, Taipei Medical University, Taipei, Taiwan; 5 Graduate Institute of Pharmacology, College of Medicine, National Taiwan University, Taipei, Taiwan; 6 Department of Medicine, University of British Columbia, Vancouver, British Columbia, Canada; 7 Division of General Surgery, Department of Surgery, Landseed Hospital, Taoyuan, Taiwan; 8 Department of Pharmacology, School of Medicine, College of Medicine, Taipei Medical University, Taipei, Taiwan; China Medical University, TAIWAN

## Abstract

Elevated serum interleukin-6 (IL-6) levels correlates with tumor grade and poor prognosis in cancer patients. IL-6 has been shown to promote tumor lymphangiogenesis through vascular endothelial growth factor-C (VEGF-C) induction in tumor cells. We recently showed that IL-6 also induced VEGF-C expression in lymphatic endothelial cells (LECs). However, the signaling mechanisms involved in IL-6-induces VEGF-C induction in LECs remain incompletely understood. In this study, we explored the causal role of focal adhesion kinase (FAK) in inducing VEGF-C expression in IL-6-stimulated murine LECs (SV-LECs). FAK signaling blockade by NSC 667249 (a FAK inhibitor) attenuated IL-6-induced VEGF-C expression and VEGF-C promoter-luciferase activities. IL-6’s enhancing effects of increasing FAK, ERK1/2, p38MAPK, C/EBPβ, p65 and STAT3 phosphorylation as well as C/EBPβ-, κB- and STAT3-luciferase activities were reduced in the presence of NSC 667249. STAT3 knockdown by STAT3 siRNA abrogated IL-6’s actions in elevating VEGF-C mRNA and protein levels. Moreover, Src-FAK signaling blockade reduced IL-6’s enhancing effects of increasing STAT3 binding to the VEGF-C promoter region, cell migration and endothelial tube formation of SV-LECs. Together these results suggest that IL-6 increases VEGF-C induction and lymphangiogenesis may involve, at least in part, Src-FAK-STAT3 cascade in LECs.

## Introduction

Metastatic spread of tumor cells remains the major cause of death in cancer patients [1, 2]. Tumor cells access to the systemic circulation and invade surrounding tissues by releasing growth factors or cytokines to stimulate angiogenesis and lymphangiogenesis [3]. Angiogenesis and lymphangiogenesis, the major routes for tumor metastasis, therefore represent rational targets for therapeutic intervention. In the last decades, there has been a remarkable progress in identifying the underlying mechanisms of tumor angiogenesis, leading to several novel anti-angiogenic agents approved by the FDA for cancer treatment [4]. However, lymphangiogenesis, in contrast to angiogenesis, has been less thoroughly explored. Similar to angiogenesis, lymphangiogenesis is defined as the process of newly-formed lymphatic capillaries from existing vessels. There have been growing evidence that lymphangiogenesis is increased in various malignancies. Patients with tumor lymphangiogenesis have less favorable outcome [5]. In addition, suppression of tumor lymphangiogenesis markedly reduced tumor metastasis in xenograft murine models [6]. Therefore, understanding the molecular signaling cascades that modulate lymphangiogenesis is mandatory for developing novel therapeutic strategies for cancer treatment.

It is generally accepted that chronic inflammation plays a pivotal role in promoting cancer development and progression [1, 7]. One of the best surrogates of chronic inflammation in the pathogenesis of tumor is interleukin-6 (IL-6), a pleiotropic inflammatory cytokine [8]. Elevated serum IL-6 levels have been found correlating with poor prognosis of patients with advanced stages of cancer [9–11]. Many lines of evidence demonstrated that IL-6 exhibits tumor-promoting actions including regulation of tumor growth [12] and chemo-resistance [10], induction of epithelial-mesenchymal transition [13] and promoting angiogenesis [14] as well as lymphangiogenesis [15], which contribute to tumor metastasis. Among known lymphangiogenic factors, vascular endothelial growth factor-C (VEGF-C) is the best-characterized and recognized as a major regulator of lymphangiogenesis. It has been believed that VEGF-C acts via VEGF receptor-3 (VEGFR-3), which is expressed dominantly on lymphatic endothelial cells (LECs) [16]. VEGF-C binds to and activates VEGFR-3 and its downstream signaling pathways that promoting LEC migration, proliferation, and survival [17]. Many lines of evidence indicated that VEGF-C plays a pivotal role in promoting tumor lymphangiogenesis and metastasis [18, 19]. Clinical studies further showed that elevated VEGF-C levels closely correlate with lymph node metastasis and poor patient prognosis [20, 21]. Shinriki et al. [15] previous demonstrated that IL-6 induces VEGF-C expression and lymphangiogenesis in human oral squamous cell carcinoma. We recently utilized a conditionally immortalized line of murine LECs (SV-LECs) [22, 23] and showed that IL-6 induces VEGF-C expression not only in tumor cells but also in LECs [24]. However, the precise mechanisms underlying IL-6-induced VEGF-C expression in LECs remain incompletely understood.

IL-6 exhibits its pleiotropic functions through IL-6 signaling complex formed by the association of IL-6, IL-6 receptor (IL-6R) and glycoprotein130 (gp130). IL-6 activates gp130-mediated cis- or trans-signaling via membrane bound or soluble form of IL-6R (sIL-6R) [25]. It has been postulated that IL-6 trans-signaling is implicated in most of dysregulated inflammatory responses leading to diseases [26]. IL-6 signaling complex activates mainly three major downstream signaling cascades including the janus tyrosine family kinase (JAK)-signal transducer and activator of transcription 3 (STAT3) signaling, the phosphoinositide 3-kinase (PI3-K) signaling, and the Ras-extracellular signal-regulated kinase (ERK) signaling [27]. We previously demonstrated that Src signaling plays a causal role in VEGF-C induction in IL-6-stimulated SV-LECs. We also noted that Src mediated IL-6-induced focal adhesion kinase (FAK) and STAT3 activation in SV-LECs [24]. However, whether FAK or STAT3 contributes to IL-6’s enhancing effects in inducing VEGF-C expression and its underlying mechanisms remain to be established. In this study, we aimed to explore whether FAK contributes to IL-6 actions in inducing VEGF-C induction and lymphangiogensis in LECs.

## Materials and Methods

### Reagents

Fetal bovine serum (FBS), DMEM, penicillin, and streptomycin were purchased from Invitrogen (Carlsbad, CA, USA). Recombinant IL-6 and soluble IL6Rα (sIL6R) were purchased from PeproTech (Rocky Hill, NJ, USA). PP2, NSC 667249 and the enhanced chemiluminescence detection kit were purchased from Millipore (Billerica, MA, USA). Antibodies against Src phosphorylated at Tyr416, FAK, FAK phosphorylated at Tyr397, p38MAPK, p38MAPK phosphorylated at Thr180/Tyr182, ERK1/2, ERK1/2 phosphorylated at Thr202/Tyr204, C/EBPβ phosphorylated at Thr235, and STAT3 phosphorylated at Tyr705 were purchased from Cell Signaling (Danvers, MA, USA). Antibodies specific for STAT3, C/EBPβ and p65 and normal IgG were purchased from Santa Cruz Biotechnology (Santa Cruz, CA, USA). Anti-mouse and anti-rabbit IgG conjugated horseradish peroxidase antibodies and antibodies against p65 phosphorylated at Ser536, VEGF-C, Src and α-tubulin were purchased from GeneTex Inc (Irvine, CA, USA). NF-κB-luc and Renilla-luc reporter constructs and Dual-Glo luciferase assay system were purchased from Promega (Madison, WI, USA). Turbofect^TM^ in vitro transfection reagent was purchased from Upstate Biotechnology (Lake Placid, NY, USA). Murine VEGF-C promoter sequence (−370/+1) cloned into pGL4-basic vector (pGL4-VEGF-C promoter-luc-370) was constructed as described previously [24]. The C/EBP-luc reporter construct was kindly provided by Dr Kjetil Tasken (University of Oslo, Oslo, Norway). The STAT3-luc (p4xM67-tk-Luc, Addgene plasmid 8688) reporter construct containing four copies of STAT-binding site as described previously [28] was provided by Dr. James E. Darnell Jr. (Laboratory of Molecular Cell Biology, The Rockefeller University, New York, USA). All materials for immunoblotting were purchased from GE Healthcare (Little Chalfont, UK). Negative control siRNA, mouse STAT3 siRNA and all other chemicals were obtained from Sigma-Aldrich (St. Louis, MO, USA).

### Cell culture

The murine LEC line SV-LEC was kindly provided by Dr. J.S. Alexander (Shreveport, LA, USA) [22]. SV-LECs were maintained in streptomycin (100 μg/ml), penicillin G (100 U/ml) and 10% FBS containing DMEM medium in a humidified 37°C incubator.

### Immunoblotting

Immunoblotting was performed as described previously [29]. Briefly, cells were lysed in an lysis buffer (140 mM NaCl, 2 mM phenylmethanesulfonylfluooride (PMSF), 0.5% NP-40, 10 mM Tris (pH 7.0), 0.2 mM leupeptin, and 0.05 mM pepstatin A. Samples with equal amounts of protein were subjected to SDS-PAGE and transferred onto an nitrocellulose membrane (NC paper). After blocking using 5% non-fat milk containing TBST buffer, proteins were visualized by incubating with specific primary antibodies for 2 h followed by horse radish peroxidase-conjugated secondary antibodies for another 1 h. The enhanced chemiluminescence detection kit (Millipore, Billerica, MA, USA) was used to detect immunoreactivity according to the manufacturer's instructions. Quantitative data was obtained using a computing densitometer with a scientific imaging system (Biospectrum AC System, UVP, Upland, CA, USA).

### Gene Suppression

For STAT3 suppression, predesigned siRNA targeting the mouse STAT3 was purchased from Sigma-Aldrich (St. Louis, MO, USA). The siRNA oligonucleotide targeting the coding regions of mouse STAT3 was as follows: STAT3 siRNA, 5′-guugaauuaucagcuuaaa-3′. The negative control siRNA comprising a 21-bp scrambled sequence with 3′dT overhangs was also purchased from Sigma-Aldrich (St. Louis, MO, USA).

### Cell Transfection

SV-LECs were transfected with siRNAs or different constructs as described below using Turbofect transfection reagent (Millipore, Billerica, MA, USA) according to the manufacturer's instructions. For the reporter assay, cells were transfected with pGL4-VEGF-C-luc-370 [24], C/EBPβ-luc, κB-luc or STAT3-luc plus Renilla-luc. For immunoblotting or RT-PCR analysis, cells were transfected with negative siRNA or mouse STAT3 siRNA. Using the same transfection protocol, we assessed the transfection efficiency based on pEGFP, a green fluorescence protein expression vector using flow-cytometric analysis with FACScan and Cellquest program (Becton Dickinson). Transfection efficiency is defined as the percentage of cells expressing green fluorescence. The compiled results show a transfection rate is approximately 40% (N = 3) (S1 Fig).

### Dual luciferase reporter assay

Cells with or without treatments were harvested. The luciferase activity was then determined using a Dual-Glo luciferase assay system kit (Promega, Madison, WI, USA) according to manufacturer’s instructions, and was normalized on the basis of renilla luciferase activity as described previously [29].

### Reverse-transcription polymerase chain reaction (RT-PCR)

RT-PCR analyses were performed as described previously [24]. Primers used for amplifying the mouse VEGF-C and GAPDH fragments were as follows: VEGF-C, sense: 5'-AGCCAACAGGGAATTTGATG-3' and antisense: 5'-CACAGCGGCATACTTCTTCA-3'; GAPDH, sense: 5’-CCTTCATTGACCTCAACTAC-3’ and antisense: 5’-GGAAGGCCATGCCAGTGAGC-3’. GAPDH was used as the internal control. The PCR was performed with the following conditions: 30 cycles of a 30-s denaturation step at 94°C, a 30-s annealing step at 57°C, and a 45-s extension step at 72°C to amplify VEGF-C and GAPDH cDNA. The amplified fragment sizes for mouse VEGF-C and GAPDH were 239 and 594 bp, respectively. PCR products were subjected to agarose gel electrophoresis, stained with ethidium bromide, and visualized by ultraviolet illumination.

### Enzyme-linked Immunosorbent Assay (ELISA)

After treatment as indicated, the cell culture medium was removed and stored at -80°C until the assay. The concentration of VEGF-C in the culture medium was quantified using a VEGF-C ELISA kit (Cloud-Clone Corp, Houston, TX, USA), according to the manufacturer’s protocol.

### Cell migration assay (Wound-healing cell scratch assay)

SV-LECs were grow to confluence in 12-well tissue culture plates. Cells were then starved with serum-free DMEM medium for 16 h. After starvation, monolayer SV-LECs were wounded by scratching with pipette tips and washed with PBS. DMEM medium was added into the wells in the presence of absence of IL-6/sIL-6R (20 ng/ml). After 24 h treatment, cells were photographed under an inverted contrast phase microscope (Nikon, Japan). The rate of cell migration was quantified by counting the number of migrated cells in the scratch area.

### Matrigel tube formation assay

Matrigel, a basement membrane matrix (Becton Dickinson, Mountain View, CA, USA), was polymerized at 37°C for 30 min. SV-LECs suspended in DMEM medium in the presence or absence of IL-6/sIL-6R (20 ng/ml) were seeded onto the Matrigel. After 3 h, capillary-like tubes were photographed under an inverted contrast phase microscope (Nikon, Japan). The total length of the tubes was measured using pen type digital meter (KOIZUMI Sokki Mfg. Co., Ltd, Niigata, Japan) for the quantification of tube formation.

### Chromatin immunoprecipitation (ChIP) assay

A ChIP assay was performed as described previously [24]. Purified DNA was used for the PCR in 50 μL reaction mixture. The 170-bp VEGF-C promoter fragment between -198 and -367 (contains putative STAT3 binding sites) was amplified using the primer pair, sense: 5’-cgg gac gag tgg aac atc-3’ and antisense: 5’-agg tac gag cct cac agg aa-3’, in 30 cycles of PCR. This was done at 95°C for 30 s, 56°C for 30 s, and finally 72°C for 45 s. PCR products were subjected to agarose gel electrophoresis, stained with ethidium bromide, and visualized by ultraviolet illumination.

### Statistical analysis

Results are presented as the mean ± S.E. from at least three independent experiments. One-way analysis of variance (ANOVA) was followed by the Newman-Keuls test, when appropriate, to determine the statistical significance of the difference between means. A *p* value of < 0.05 was considered statistically significant.

## Results

### FAK inhibitor NSC 667249 reduced IL-6/sIL-6R-induced VEGF-C expression in SV-LECs

**Fig 1:**
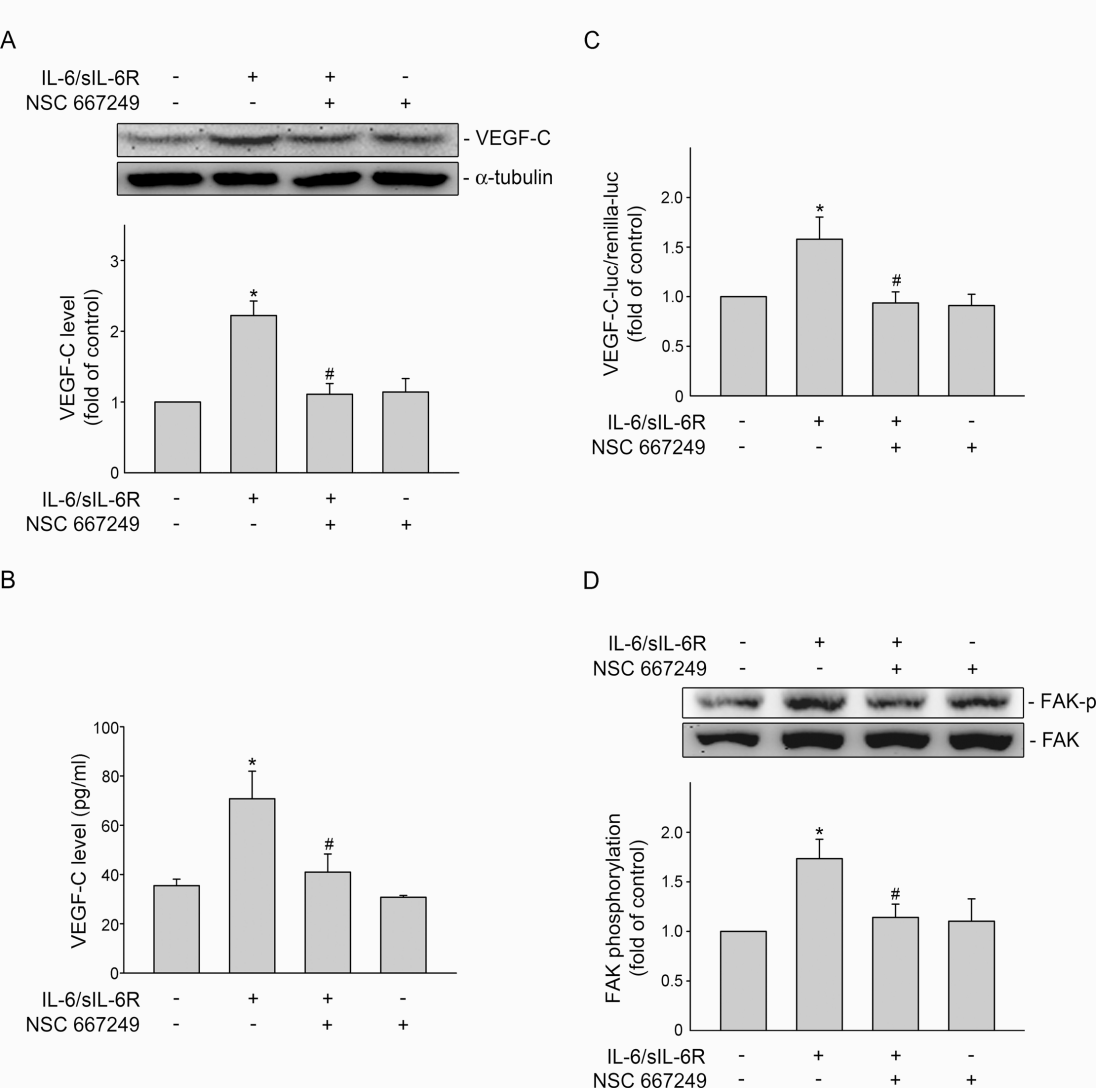
FAK contributed to IL-6/sIL-6R-induced VEGF-C expression in SV-LECs. (A) Cells were pretreated with the vehicle or NSC 667249 (0.3 μM) for 30 min before treatment with IL-6 plus sIL6R (20 ng/ml) for another 24 h. The VEGF-C level and was then determined by immunoblotting. Each column represents the mean ± S.E.M. of eight independent experiments. (B) After treatment as described in (A), cell culture media were collected and VEGF-C in the media was quantified using a ELISA kit. Each column represents the mean ± S.E.M. of four independent experiments. (C) Cells were transiently transfected with VEGF-C promoter-luc-370 and renilla-luc for 48 h. After transfection, cells were treated with vehicle or NSC 667249 (0.3 μM) for 30 min, followed by treatment with IL-6 plus sIL6R (20 ng/ml) for another 24 h. Luciferase activity was then determined as described in the “Materials and Methods” section. Data represent the mean ± S.E.M. of three independent experiments performed in duplicate. (D). Cells were pretreated with the vehicle or NSC 667249 (0.3 μM) for 30min followed by treatment with IL-6 plus sIL6R (20 ng/ml) for another 30 min. The extent of FAK phosphorylation was then determined by immunoblotting. Each column represents the mean ± S.E.M. of four independent experiments. *p<0.05, compared to the control group; ^#^ p<0.05, compared to the vehicle-treated group in the presence of IL-6 plus sIL6R.

To determine whether FAK signaling contributes to VEGF-C induction in IL-6/sIL-6R-stimulated SV-LECs, a pharmacological FAK inhibitor NSC 667249 was used. As shown in Fig 1A, treatment of SV-LECs with IL-6/sIL-6R (20 ng/ml) for 24 h caused a significant increase in VEGF-C protein levels. After 24 h of IL-6/sIL-6R exposure, VEGF-C released into cell culture medium was also increased (Fig 1B). However, IL-6/sIL-6R-induced VEGF-C induction (Fig 1A) and VEGF-C release (Fig 1B) were significantly reduced in the presence of NSC 667249. We next performed reporter assay with murine VEGF-C promoter-luc reporter construct [24] to explore whether NSC 667249 attenuation of VEGF-C induction at transcriptional level. As shown in Fig 1C, NSC 667249 significantly reduced IL-6/sIL-6R’s enhancing effects in increasing VEGF-C promoter luciferase activity in SV-LECs. Confirming the inhibitory effects of NSC 667249, a marked reduction in IL-6/sIL-6R-induced FAK phosphorylation was observed in SV-LECs exposed to NSC 667249 (Fig 1D). These results suggest that IL-6/sIL-6R-induced VEGF-C expression is attributed to FAK signaling in SV-LECs.

### FAK mediated ERK1/2 and p38MAPK phosphorylation in IL-6/sIL-6R-stimulated SV-LECs

**Fig 2:**
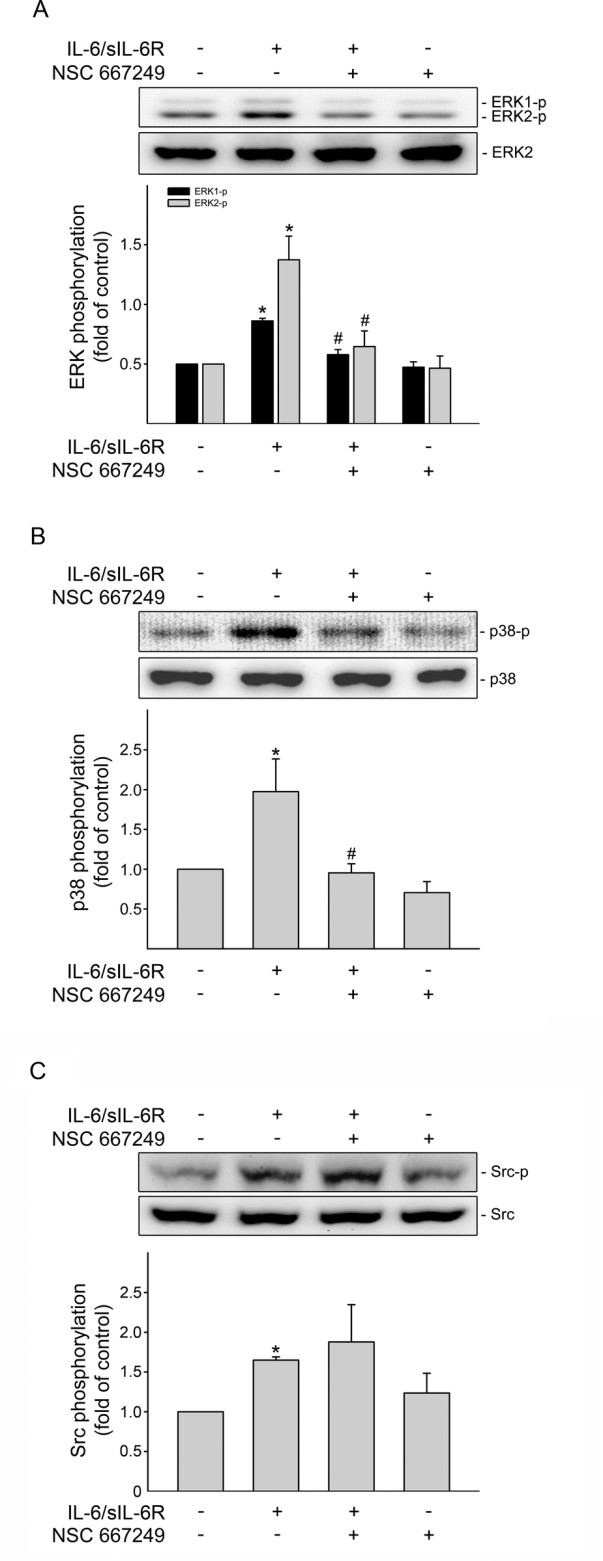
FAK mediated IL-6/sIL-6R-induced ERK1/2 and p38MAPK phosphorylation in SV-LECs. Cells were pretreated with the vehicle or NSC 667249 (0.3 μM) for 30min followed by treatment with IL-6 plus sIL6R (20 ng/ml) for another 30 min. The extent of ERK1/2 (A), p38MAPK (B) or Src (C) phosphorylation was then determined by immunoblotting. Each column represents the mean ± S.E.M. of five independent experiments. *p<0.05, compared to the control group; ^#^ p<0.05, compared to the vehicle-treated group in the presence of IL-6 plus sIL6R.

As previous report that ERK1/2 and p38MAPK are implicated in induction of VEGF-C [24], we next sought to determine whether IL-6/sIL-6R-induced ERK1/2 and p38MAPK phosphorylation involves FAK signaling. As shown in Fig 2A, FAK signaling blockade by NSC 667249 significantly suppressed ERK1/2 phosphorylation in SV-LECs exposed to IL-6/sIL-6R. Similarly, NSC 667249 also inhibited IL-6/sIL-6R-induced p38MAPK phosphorylation (Fig 2B). In contrast, the phosphorylation status of Src was not altered despite the presence of NSC 667249 in IL-6/sIL-6R-stimulated SV-LECs (Fig 2C). These findings together suggest that Src-mediated FAK activation may occur upstream of ERK1/2 and p38MAPK signaling cascades in SV-LECs after IL-6/sIL-6R exposure.

### FAK contributed to IL-6/sIL-6R-induced C/EBPβ, p65 and STAT3 phosphorylation in SV-LECs

**Fig 3:**
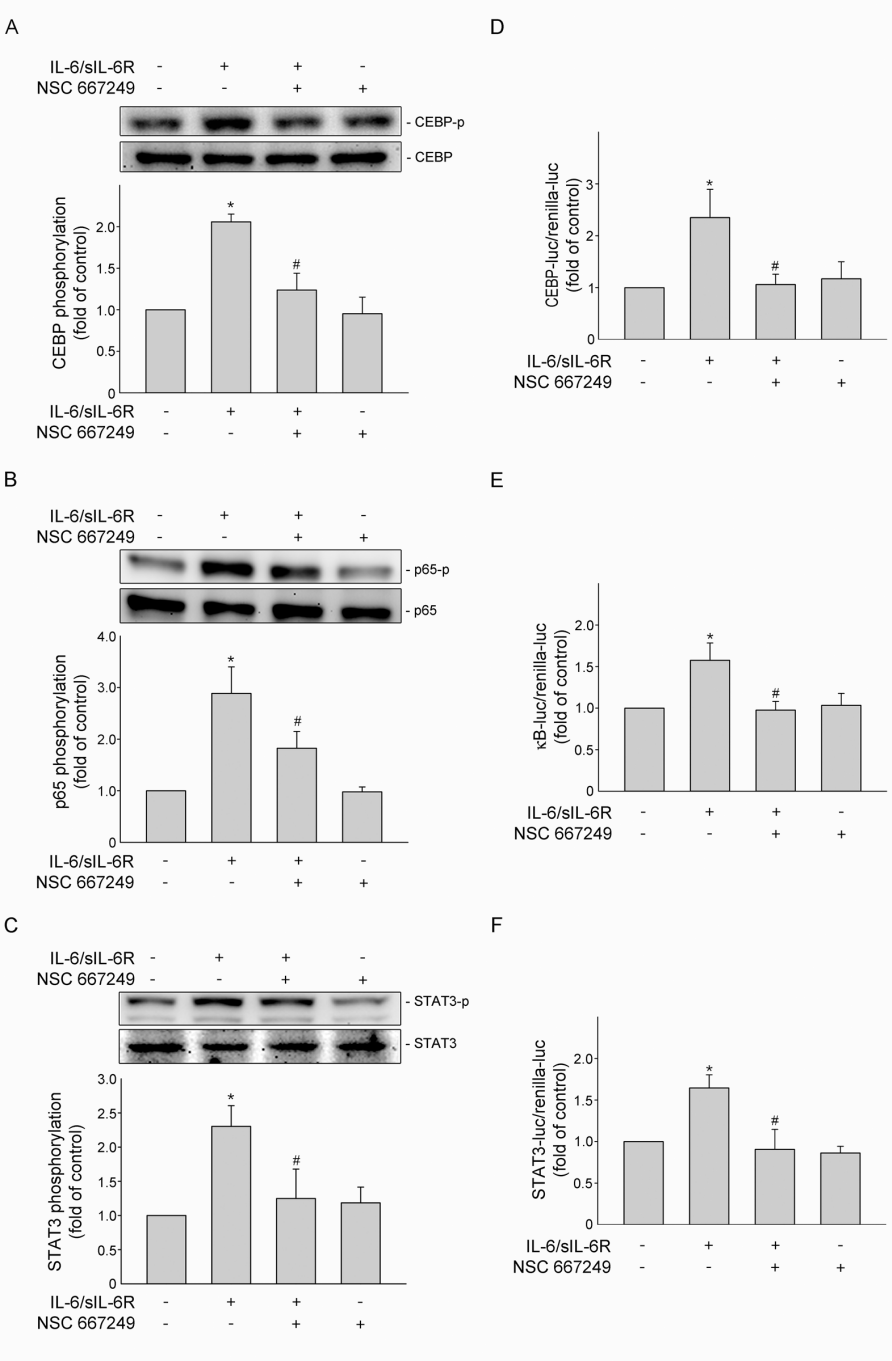
FAK contributed to IL-6/sIL-6R-induced C/EBPß, NF-κB and STAT3 activation in SV-LECs. Cells were pretreated with the vehicle or NSC 667249 (0.3 μM) for 30min followed by treatment with IL-6 plus sIL6R (20 ng/ml) for another 30 min. The extent of C/EBPß (A), p65 (B) or STAT3 (C) phosphorylation was then determined by immunoblotting. Each column represents the mean ± S.E.M. of five independent experiments. *p<0.05, compared to the control group; ^#^ p<0.05, compared to the vehicle-treated group in the presence of IL-6 plus sIL6R. Cells were transfected with C/EBPß (D), NF-κB-luc (E) or VEGF-C promoter-luc-370 (F) plus renilla-luc for 48 h. After transfection, SV-LECs were pretreated with the vehicle or NSC 667249 (0.3 μM) for 30min followed by treatment with IL-6 plus sIL6R (20 ng/ml) for another 24 h. The luciferase activity was then determined. Each column represents the mean ± S.E.M. of at least four independent experiments. *p<0.05, compared to the control group; ^#^ p<0.05, compared to the vehicle-treated group in the presence of IL-6 plus sIL6R.

Transcription factors including NF-κB and C/EBPß play pivotal roles in transcriptional induction of VEGF-C [24]. It has been shown that C/EBPß phosphorylation promotes its transcriptional activity [30]. Moreover, the phosphorylation of NF-κB subunit p65 accounts for its transcriptional activity [31]. We therefore investigated whether NSC 667249 affects phosphorylation status of C/EBPß and p65 in IL-6/sIL-6R-stimulated SV-LECs. Results from immunoblotting analysis demonstrated that NSC 667249 significantly reduced C/EBPß (Fig 3A) and p65 (Fig 3B) phosphorylation in SV-LECs exposed to IL-6/sIL-6R. We also examined whether phosphorylation status of STAT3 is altered in the presence of NSC 667249. As shown in Fig 3C, NSC 667249 significantly inhibited IL-6/sIL-6R-induced STAT3 phosphorylation. Results from reporter assays further showed that NSC 667249 attenuated IL-6/sIL-6R’s effects of increasing C/EBPß- (Fig 3D), NF-κB- (Fig 3E) and STAT3-luciferase (Fig 3F) activities. Together these observations suggest that FAK may active downstream transcriptional factors including C/EBPß, NF-κB and STAT3 in IL-6/sIL-6R-stimulated SV-LECs.

### STAT3 plays a causal role in VEGF-C induction in IL-6/sIL-6R-stimulated SV-LECs

**Fig 4:**
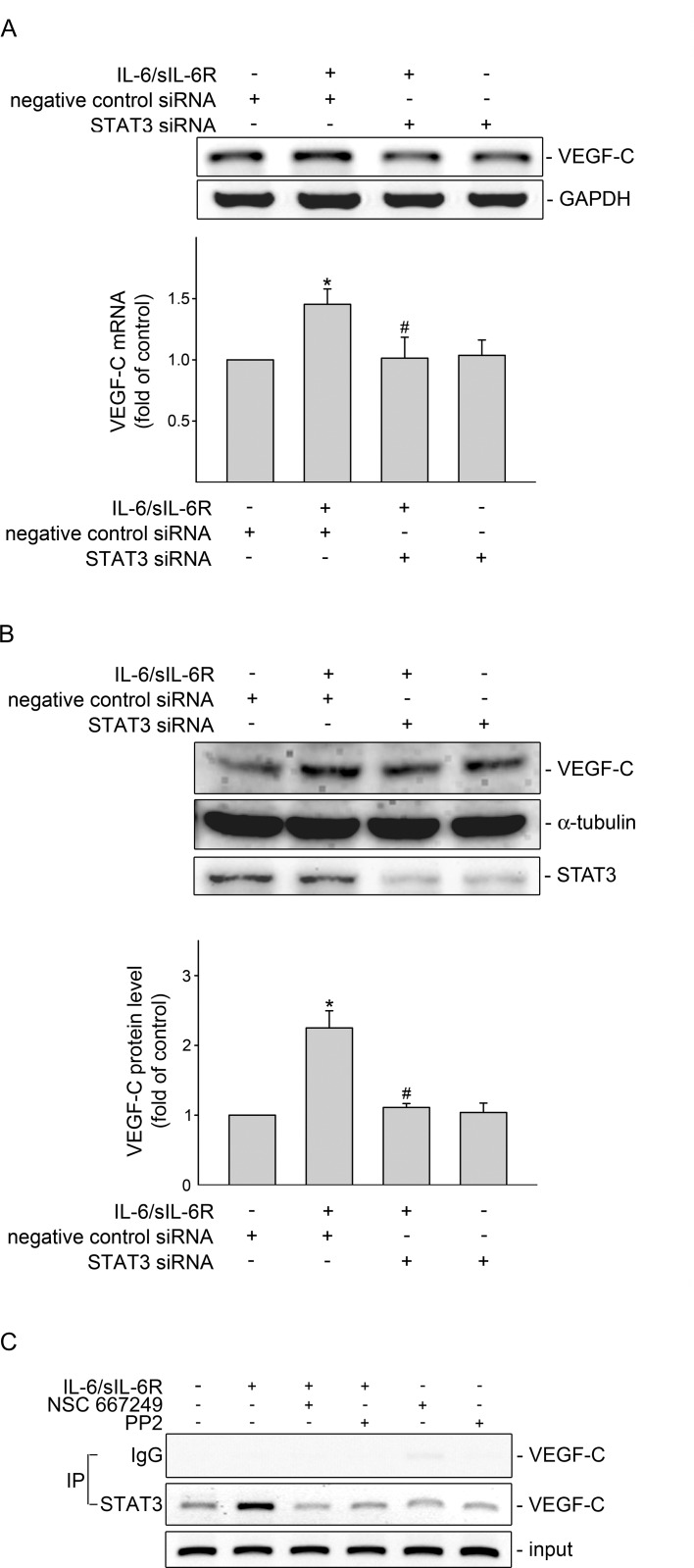
STAT3 siRNA suppressed IL-6/sIL-6R-induced VEGF-C expression in SV-LECs. (A) Cells were transiently transfected with negative control siRNA or STAT3 siRNA for 48 h. After transfection, cells were treated with IL-6 plus sIL6R (20 ng/ml) for another 6 h. The extent of VEGF-C mRNA was analyzed by RT-PCR as described in the “Materials and Methods” section. Each column represents the mean ± S.E.M. of five independent experiments. *p<0.05, compared to the control group; ^#^ p<0.05, compared to the vehicle-treated group in the presence of IL-6 plus sIL6R. (B) Cells were transiently transfected with negative control siRNA or STAT3 siRNA for 48 h. After transfection, cells were treated with IL-6 plus sIL6R (20 ng/ml) for another 24 h. The VEGF-C protein level was then determined by immunoblotting. Each column represents the mean ± S.E.M. of four independent experiments. * p<0.05, compared to the control group; ^#^ p<0.05, compared to the vehicle-treated group in the presence of IL-6 plus sIL6R. (C) Cells were pretreated with the vehicle, PP2 (1 μM) or NSC 667249 (0.3 μM) for 30 min followed by treatment with IL-6 plus sIL6R (20 ng/ml) for another 4 h. The ChIP assay was performed as described in the ‘‘Materials and methods” section. Typical traces representative of three independent experiments with similar results are shown. The VEGF-C promoter region (-367/-198) was detected in the cross-linked chromatin sample before immunoprecipitation (bottom panels of the chart, Input, positive control).

To confirm whether STAT3 contributes to IL-6/sIL-6R’s actions in inducing VEGF-C expression, siRNA strategy was used to silence STAT3 expression in SV-LECs. As shown in Fig 4A, STAT3 siRNA significantly reduced VEGF-C mRNA expression in IL-6/sIL-6R-stimulated SV-LECs. IL-6/sIL-6R-increased VEGF-C protein level was also suppressed by STAT3 siRNA (Fig 4B). In addition, basal STAT3 level in SV-LECs was markedly reduced in cells transfected with STAT3 siRNA (Fig 4B). These results suggest that STAT3 is responsible for VEGF-C induction in SV-LECs exposed to IL-6/sIL-6R. Results from TFSEARCH analysis (Yutaka Akiyama: ‘‘TFSEARCH: Searching Transcription Factor Binding Sites”; http://www.rwcp.or.jp/papia/) [32] showed that putative consensus sequence for STAT3 is included in the 5’-flanking region (-367 to -198) of the murine VEGF-C gene3. To determine whether IL-6/sIL-6R increases STAT3 binding to the endogenous VEGF-C promoter region (-367 to -198), a chromatin immunoprecipitation (ChIP) analysis was performed. As shown in Fig 4C. IL-6/sIL6R increased the recruitment of STAT3 to the VEGF-C promoter region (-367/-198). However, this enhancing effect of IL-6/sIL6R was abolished in the presence of PP2 (a Src inhibitor) or NSC 667249 (Fig 4C). These observations suggest that IL-6/sIL-6R-induced STAT3 activation and VEGF-C expression through Src-FAK signaling cascade in SV-LECs.

### Src-FAK signaling blockade reduced IL-6/sIL-6R-induced cell migration and tube formation of SV-LECs

**Fig 5:**
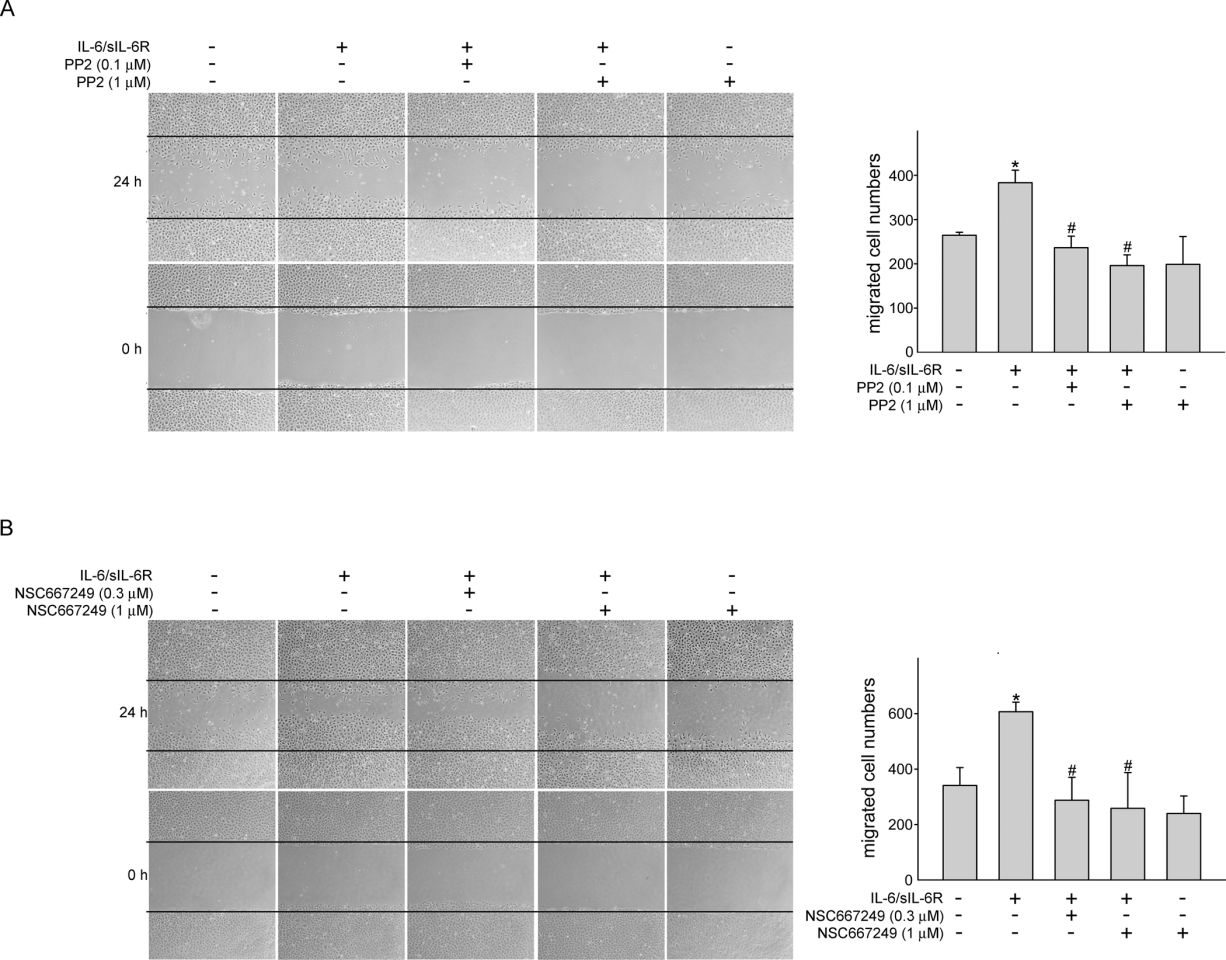
Src-FAK signaling blockade reduced IL-6/sIL-6R-induced SV-LEC migration. SV-LECs were starved in DMEM medium without FBS for 16 h. After starvation, cells were pretreated with PP2 (A) or NSC 667249 (B) at indicated concentrations followed by the stimulation with IL-6 plus sIL6R (20 ng/ml) for another 24 h. Cell migration was determined as described in the ‘‘Materials and methods” section. Each column represents the mean ± S.E.M. of five independent experiments * p<0.05, compared to the control group; ^#^ p<0.05, compared to the vehicle-treated group in the presence of IL-6 plus sIL6R.

**Fig 6:**
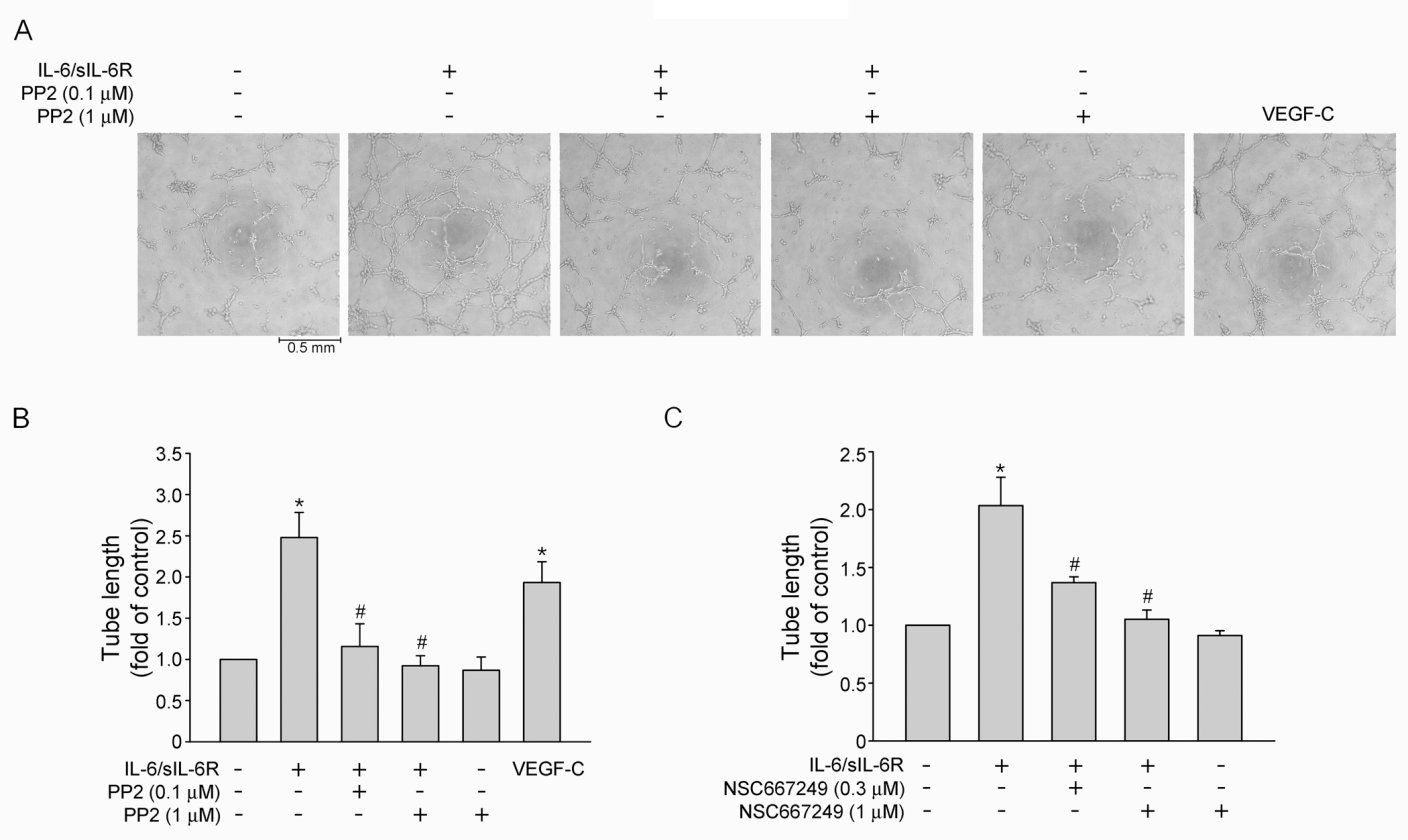
Src-FAK signaling blockade suppressed IL-6/sIL-6R-induced tube formation of SV-LECs. (A) SV-LECs were seeded on Matrigel in the presence of VEGF-C (50 ng/ml) or IL-6 plus sIL6R (20 ng/ml) with or without PP2. Cells were then photographed under phase-contrast after 3 h. A representative microscopic phenotype of the formed tubes is shown (N = 3). (B) Total tube length of the formed capillary-like tube under different treatments as described in (A) was also measured using pen type digital meter. Each column represents the mean ± S.E.M. of three independent experiments. (C) SV-LECs were seeded on Matrigel in the presence of IL-6 plus sIL6R (20 ng/ml) with or without NSC 667249. Cells were then photographed under phase-contrast after 3 h. Total tube length of the formed capillary-like tube under different treatments was measured using pen type digital meter. Each column represents the mean ± S.E.M. of three independent experiments. * p<0.05, compared to the control group; ^#^ p<0.05, compared to the vehicle-treated group in the presence of IL-6 plus sIL6R.

Similar to angiogenesis, sprouting from existing lymphatic vessels, migration and forming a capillary-like structure are required for lymphamgiogenesis [33, 34]. Cell migration assay and tube-formation assay were employed to explore whether IL-6 exhibits lymphangiogenic properties. As shown in Fig 5A treatment of SV-LECs with IL-6/sIL-6R (20 ng/ml) for 24 h significantly increased cell motility as determined by counting migrated cells. However, Src or FAK signaling blockade using PP2 (Fig 5A) or NSC 667249 (Fig 5B) markedly reduced IL-6/sIL-6R’s enhancing effects on cell migration. In addition, SV-LECs became elongated and formed capillary-like structures after exposure to IL-6/sIL-6R for 3 h as determined by two-dimensional matrigel tube formation assay (Fig 6A). Total tube length was quantified to represent the degree of tube formation. Similar to the results derived from cell migration assay, PP2 (Fig 6B) and NSC 667249 (Fig 6C) also significantly suppressed IL-6/sIL-6R-induced tube formation of SV-LECs. Taken together, these results indicate that IL-6/sIL-6R may activate, at least in part, Src-FAK signaling pathway, leading to lymphangiogenesiss.

## Discussion

In addition to angiogenesis, it is believed that VEGF-C-associated lymphangiogenesis also contributes to tumor metastasis [19, 35]. VEGF-C signaling thus represents a rational target for limiting lymphangiogenesis and metastasis. Targeting VEGF-C-VEGFR3 signaling has recently been shown benefit in reducing tumor lymphatic metastasis [36, 37]. However, reducing tumor cells- or LECs-derived VEGF-C might be another potential strategy for therapeutic intervention, although the signaling mechanisms involved in VEGF-C induction remain largely unclear. Inflammation has long been associated with tumor progression. It appears that IL-6, of all pro-inflammatory cytokines, plays a major role in modulating tumor progression [8]. Pro-inflammatory cytokines including tumor necrosis factor-α [38], RNAKL [39] and IL-6 [15] were reported to induce VEGF-C induction in macrophages, astrocytes, fibroblasts, osteoclasts, as well as in tumor cells. We showed that IL-6 also induced VEGF-C expression in SV-LECs [24], the mechanism of which was further explored in this study. We demonstrated in this study that IL-6-induced VEGF-C induction and lymphangiogenesis may involve the activation of Src-FAK-STAT3 signaling in SV-LECs.

Src is often found deregulated in various human cancers. Src promotes tumor progression by inducing proliferation, migration, as well as angiogenesis via its downstream signaling molecules such as MAPKs and FAK [40–43]. Ou et al [44] have shown that Src and FAK signaling pathways participate in colorectal cancer lymphangiogenesis. We previously demonstrated that Src activation is required for IL-6/sIL-6R-induced VEGF-C induction in SV-LECs. We also found that FAK activation is induced by Src in IL-6/sIL-6R-stimulated SV-LECs [24]. However, the role FAK in modulating lymphangiogenesis remains incompletely understood. We noted in this study that pharmacological FAK inhibition prevented IL-6/sIL-6R-induced ERK1/2 and p38MAPK activations. We also noted that FAK activation contributes to VEGF-C induction in SV-LECs after IL-6/sIL-6R exposure. These observations together suggest that FAK mediates p38MAPK and ERK1/2 activation and subsequent signaling events in IL-6/sIL-6R-stimulated SV-LECs. Moreover, Liu et al [45] demonstrated that FAK is required for JNK1/2 activation in fibroblasts in response to inflammatory stimuli. JNK1/2 activation also contributes to IL-6’s actions in vascular endothelial cells [46]. It is likely that IL-6/sIL-6R may cause FAK-medicated JNK1/2 activation in SV-LECs. Further investigations are needed to clarify whether IL-6/sIL-6R-induced VEGF-C induction involves JNK1/2-associated signaling pathways and the underlying mechanisms in SV-LECs.

Persistent activation of STAT3 is commonly observed in tumor tissues [47] and it is the core transcription factor that links inflammation to cancer [48]. Similar to the observations that STAT3 contributes to VEGF-C induction in vascular endothelial cells [49], we noted in this study that IL-6/sIL-6R-induced VEGF-C induction also involved STAT3 in SV-LECs. It is believed that IL-6’s pleiotropic tumor-promoting activities are attributable to JAK2-STAT3 signaling cascade [50]. However, tyrosine kinases other than JAK2 may also contribute to IL-6-induced STAT3 activation. We showed in the present study that STAT3 phosphorylation was causally related to FAK activation in SV-LECs after IL-6/sIL-6R exposure. In addition, c-Src [24], p38MAPK [51] and ERK1/2 [52] have also been shown to mediate STAT3 activation. The crosstalk between JAK2, Src and FAK signaling links to STAT3 activation remains to be resolved in SV-LECs. It appears, at least in part, that activation of FAK by IL-6/sIL-6R results in ERK1/2 and p38MAPK phosphorylation and subsequent STAT3 activation, as well as VEGF-C induction in SV-LECs.

The promoter region (-370~+1) of the murine *vegf-c* gene also contains Sp1, NF-κB, and C/EBPβ binding sites in addition to STAT3. Several lines of evidence demonstrated that activation of Sp1 is mediated by ERK1/2 or p38MAPK signaling pathway [53, 54]. It raises the possibility that Sp1 may also contribute to VEGF-C induction in IL-6/sIL-6R-stimulated SV-LECs. Moreover, we recently established that IL-6/sIL-6R activated p38MAPK or ERK1/2 resulting in NF-κB and C/EBPβ activation and VEGF-C induction in SV-LECs [24]. Together, these findings support the contention that IL-6/sIL-6R induced VEGF-C induction attributes to not only STAT3, but also Sp1, NF-κB and C/EBPβ in SV-LECs. The precise mechanisms by which IL-6/sIL-6R activates these transcription factors remain to be investigated. It is likely that activation of these signaling cascades may culminate in VEGF-C induction in SV-LECs. In addition, it is reported that STAT3 interacts physically and functionally with C/EBPβ [55]. Snyder et al [56] recently demonstrated that STAT3-NF-κB complex is essential for target gene expression in metastatic breast cancer cells in response to IL-6. Moreover, independent binding of STAT3 and NF-κB to the promoter region may synergistically enhance its target gene expression [48, 57]. Further investigations are needed to characterize whether STAT3 cooperates with NF-κB, C/EBPβ, Sp1 or other transcription factors in inducing VEGF-C expression in IL-6/sIL-6R-stimulated SV-LECs. Regulation of cellular VEGF-C may also occur at not only transcriptional, but also post-transcriptional level via microRNAs [58]. Rokavec et al [59] demonstrated that IL-6-STAT3 signaling regulates microRNAs to promote colorectal cancer cell endothelial-mesenchymal transition (EMT) and tumor metastasis. It is likely that VEGF-C induction by IL-6/sIL-6R may not only involve transcriptional, but also post-transcriptional mechanisms. It is worthy to explore the role of microRNAs in VEGF-C induction in IL-6/sIL-6R-stimulated SV-LECs.

**Fig 7:**
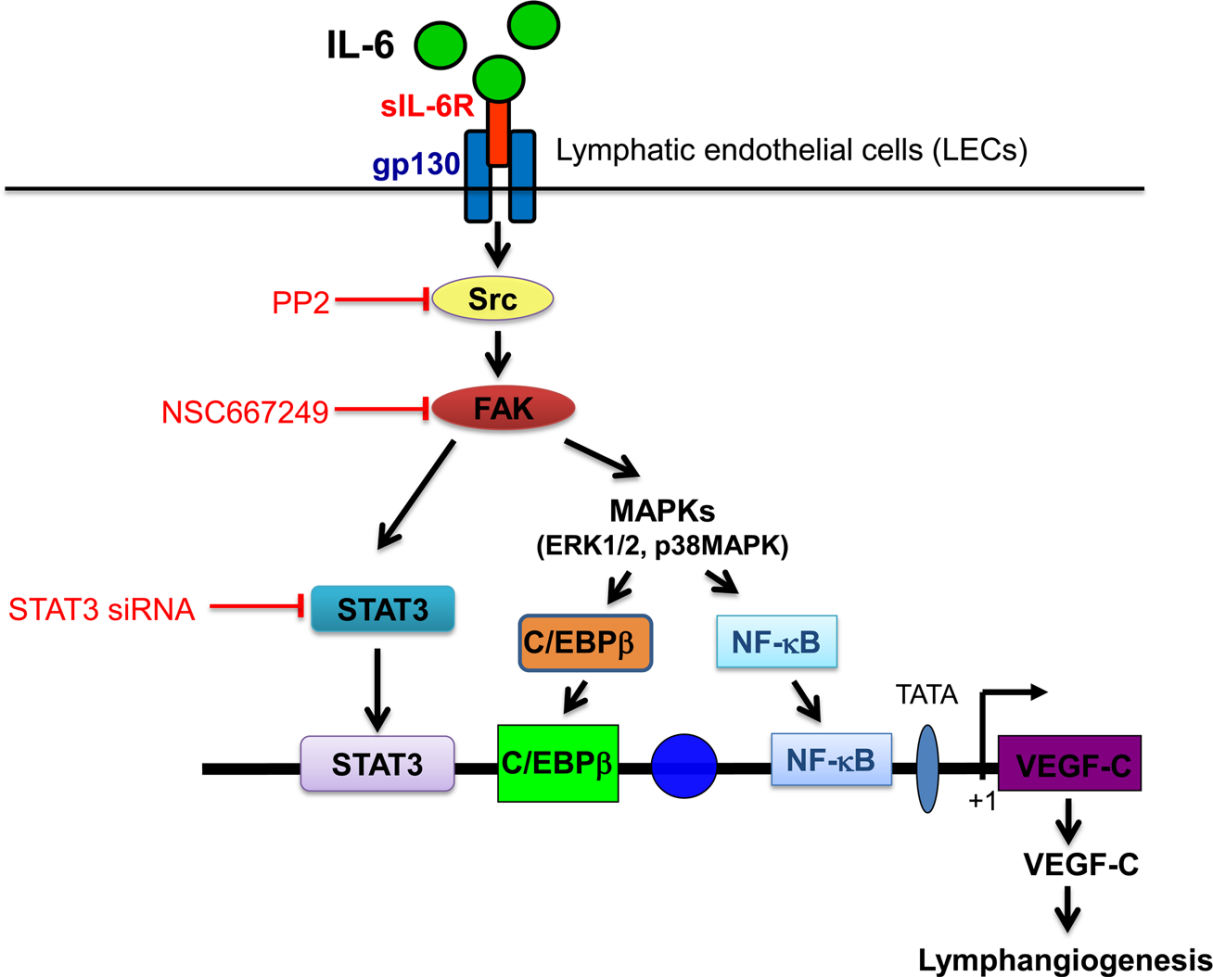
Schematic summary of IL-6-induced VEGF-C expression in SV-LECs. IL-6/sIL-6R activates the Src-FAK signaling cascade, leading to STAT3, C/EBPβ or NF-κB activation and subsequent VEGF-C expression SV-LECs.

In conclusion, we demonstrated in this study that FAK plays a causal role in IL-6/sIL-6R activation of the ERK1/2, p38MAPK and STAT3 signaling, leading to VEGF-C expression in SV-LECs (Fig 7). The present study delineates, at least in part, the signaling pathways involved in IL-6/sIL-6R-induced VEGF-C expression and lymphangiogenesis in SV-LECs. A better understanding of these underlying mechanisms may help in developing novel therapeutic strategies to reduce lymphangiogenesis and tumor metastasis.

## Supporting Information

S1 Fig Transfection efficiency in SV-LECs. SV-LECs were transfected with control vector (pcDNA) or a green fluorescence protein expression vector pEGFP as described in the “Materials and methods” section. After transfection, cells were harvested and resuspended in PBS. Green fluorescence derived from successful transfected cells were determined by flow-cytometric analysis with FACScan and Cellquest program (Becton Dickinson). Transfection efficiency is defined as the percentage of cells expressing green fluorescence (GF). The compiled results show a transfection rate is approximately 40% (N = 3).
(PDF)

## Abbreviations

CHIP: Chromatin immunoprecipitation (ChIP)
ERK: extracellular signal-regulated kinase
FAK: focal adhesion kinase
JAK: janus tyrosine family kinas
IL-6: interleukin-6
LECs: lymphatic endothelial cells
PI3-K: phosphoinositide 3-kinase
sIL-6R: soluble form of IL-6 receptor
STAT3: signal transducer and activator of transcription 3
VEGF-C: vascular endothelial growth factor-C (VEGF-C)
VEGFR-3: VEGF receptor-3
