# Supplementary material for: Interleukin-6 Induces Vascular Endothelial Growth Factor-C Expression via Src-FAK-STAT3 Signaling in Lymphatic Endothelial Cells
Source: PLoS One. 2016 Jul 6;11(7):e0158839. doi: 10.1371/journal.pone.0158839 (PMC4934912; doi:10.1371/journal.pone.0158839)
Supplement: S1 Fig — SV-LECs were transfected with control vector (pcDNA) or a green fluorescence protein expression vector pEGFP as described in the “Materials and methods” section. After transfection, cells were harvested and resuspended in PBS. Green fluorescence derived from successful transfected cells were determined by flow-cytometric analysis with FACScan and Cellquest program (Becton Dickinson). Transfection efficiency is defined as the percentage of cells expressing green fluorescence (GF). The compiled results show a transfection rate is approximately 40% (N = 3). (PDF) [file pone.0158839.s001.pdf]

S1 Fig

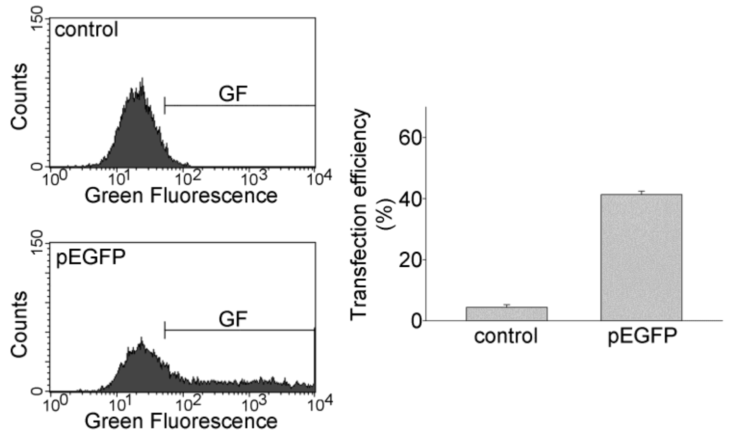

S1 Fig. Transfection efficiency in SV-LECs

SV-LECs were transfected with control vector (pcDNA) or a green fluorescence protein expression vector pEGFP as described in the “Materials and methods” section. After transfection, cells were harvested and resuspended in PBS. Green fluorescence derived from successful transfected cells were determined by flow-cytometric analysis with FACScan and Cellquest program (Becton Dickinson). Transfection efficiency is defined as the percentage of cells expressing green fluorescence (GF). The compiled results show a transfection rate is approximately 40 % (N=3).
